# Artificial intelligence and human translation: A contrastive study based on legal texts

**DOI:** 10.1016/j.heliyon.2024.e28106

**Published:** 2024-03-14

**Authors:** Ahmed Mohammed Moneus, Yousef Sahari

**Affiliations:** a Sana'a University, Yemen; b University of Bisha, Saudi Arabia

**Keywords:** Artificial intelligence, Translation software, Machine translation, Human translation, Legal translation

## Abstract

Artificial intelligence has advanced significantly in recent years, affecting multiple aspects of life. In particular, this has had an impact on the machine translation of texts, reducing or removing human interaction. Artificial intelligence (AI)-based translation software models have thus become widely available, and these now include Google Translate, Bing, Microsoft Translator, DeepL, Reverso, Systran Translate, and Amazon Translate. Several computer-aided translation (CAT) tools such as Memoq, Trados, Smartcat, Lokalise, Smartling, Crowdin, TextUnited, and Memsource are also available. More recently, artificial intelligence has been applied in the development of applications such as ChatGPT, ChatSonic, GPT-3 Playground, Chat GPT 4 and YouChat, which simulate conversational responses to researchers' inquiries, mimicking human interactions more directly. This study thus aimed to examine any remaining contrasts between human and AI translation in the legal field to investigate the potential hypothesis that there is now no difference between human and AI translation. The paper thus also examined concerns about whether the need for human translators will decline in the face of AI development, as well as beginning to assess whether it will ever be possible for those in the legal field to depend only on machine translation. To achieve this, a collection of legal texts from various contracts was chosen, and these pieces were both allocated to legal translators and subjected to AI translation systems. Using a contrastive methodology, the study thus examined the differences between AI and human translation, examining the strengths and weaknesses of both approaches and discussing the situations in which each approach might be most effective.

## 1 Introduction

Modern lives are heavily affected and steered by technology, including various sophisticated artificial intelligence (AI) applications, some of which have surpassed human ability in terms of completing various bureaucratic and operational tasks. However, humans still excel in tasks that require intellect and thought, based on the same excellence of human thought that has always ensured human dominance in the natural world.

There has, however, been increasing debate in recent years about how the development of artificial intelligence may affect human existence, with views in this area ranging from those who think AI will support human development to those who think it will cause multiple issues in the future. The first group, which includes Facebook founder Mark Zuckerberg, believe that the development of AI will help people in many different areas, while those who hold the opposing view believe that the growth of AI may cause society, and even humans themselves, to decline. Steven P. Koenig, for example, expressed concerns about the advancement of AI and the potential for human replacement, to the extent that he did not even completely rule out the chance that further development could result in the creation of novel forms of life.

Zuckerberg, the founder of Meta, has expressed a strong belief in the transformative power of AI, claiming that AI is perhaps the most important foundational technology of modern times [1]. Under his leadership, Meta is thus building what he claims to be the world's fastest artificial intelligence supercomputer [2]. Koenig, a scientist at the Institute for Materials Research and Engineering (IMRE), has also made significant contributions to the field of AI, including research on the electric field effect in ultrathin black phosphorus [3]. The work of these individuals thus emphasises the increasing impact of AI on multiple different facets of human existence, making it evident that AI is now not merely a form of technological progress, but instead a transformative process that reconfiguring both lives and communities.

Federspiel et al. [4] noted that AI is increasingly influencing human existence in many profound ways. It has thus been identified as a potential threat to human health and well-being due to its impacts on various social, political, economic, and security-related determinants of health. Indubitably, AI is transforming the human world, and it is thus important to ensure that this transformation is beneficial.

One specific instance of AI's impact can be seen in the hospitality industry in China: Alibaba's Future Hotel, a robotised hotel in Hangzhou, China, is a testament to the transformative power of AI. The hotel is operated entirely by industrial robots, with these taking on roles including those of waiters, cooks, and other service staff, jobs previously performed only by humans. This hotel uses AI to lower management costs and improve efficiency, replacing manual workers with automated systems and robots [5].

Choudhury and McConnell [6] have suggested that business translation systems still struggle to balance expense, quality, and time to market. In situations such as these, both machine translation (MT) and collaborative model creation and problem-solving with the assistance of the online community are thus becoming more popular.

The current study thus seeks to contrast texts translated by AI programs with those translated by humans, particularly with respect to legal documents. The study thus investigated the impact of AI on the field of translation, based on assessing its capacity to produce texts comparable to those translated by humans.

The study therefore aims.

1) To identify the differences between human and artificial intelligence translations with respect to legal documents, and
2) To evaluate the quality of AI translations of legal documents.

To achieve this, it asks the following research questions:

RQ1 What are the differences between comparable human and artificial intelligence translations in the legal field?

RQ2 To what extent can the quality of artificial intelligence be assured in terms of translating legal documents?

## 2 Literature review

### 2.1 Artificial intelligence

Artificial Intelligence (AI) has emerged as a contemporary subject of discourse within academic institutions, media outlets, and various political spheres based on the ongoing second industrial revolution. In recent years, AI development and discourse has thus influenced various sectors, including academia, industry, media, and politics. This interdisciplinary field is at the forefront of technological advancement, influencing a myriad of sectors and reshaping understanding of the world, which makes it of paramount importance for researchers to engage in rigorous exploration and critical analysis of AI within a wide range of contexts. According to Ramesh et al. [7], the development of robots can be seen as the beginning of AI development, though artificial intelligence is more broadly described by Fetzer [8] as having its origins and creation method in human invention and ingenuity, thus distinguishing artificially intelligent things from naturally intelligent things. The development of robots themselves is often attributed to the 1921 drama “R.U. R" (Rossum's Universal Robots), in which author Karel Capek popularised the word robot (robota in Czech) by writing about a factory employing bio-engineered machines to perform forced labour. Issac Asimov then cemented the word “robot" in the popular imagination of the middle of the 20th century by using it in his many short works of contemporary science fiction.

Developing intelligent technology that can mimic human ability to do tasks normally reserved for humans features many aspects. According to Russell [9], both intelligence and the successful nature of being a sound artifact are prerequisites for artificial intelligence to succeed. Based on the latter, computers have commonly been used to simulate human behaviour. However, artificial intelligence is more broadly the science of designing intelligence technology, based on developing knowledgeable computer algorithms, according to McCarthy [10]; thus, although it is related to the use of computers to develop understanding of human intellect, AI need not be limited to techniques that may be observed physically.

#### 2.2.1 AI and language

Both programming and natural languages have played a significant role in the development of artificial intelligence. According to Russell [9], computational linguistics and natural language processing can be used together to form a hybrid science between standard linguistics and the study of artificial intelligence. Understanding the subject and context is thus seen as necessary for both language comprehension and comprehending sentences generated by artificial intelligence, though this was not generally recognised until the 1960s. However, studies in linguistics, which are related in turn to decades of work on the philosophical analysis of language, motivated much of the early work in knowledge visualisation, the study of how to represent information in a way that a computer can reason with [9].

### 2.2 Artificial intelligence use in translation

A vast number of people now use smartphones and online machine translation apps to interact across language barriers, reducing the distances between cultures and linguistic systems. Yang [11] thus mentioned that, with the development of automated translation tools, a new translation concept known as artificial intelligence translation has appeared, with further scenarios emerging in new machine translation apps to offer greater equivalence to human translators; however, their opponents argue that there is still an insurmountable performance difference between the two processes [12,13].

According to Majumde et al. [14], most machine translation studies to date have concentrated on translating and assessing sentences in isolation, disregarding the context in which such sentences appear. Several advantages may thus be found by refining the translation process, including increased ease of data set generation, the development of more effective algorithmic models, and faster human evaluation. In particular, human assessment fails to reveal every translation mistake without context, potentially leading to some issues with early declarations of parity with humanity. O'Hagan [15] highlighted that ordinary Internet users may require translation, as may public groups and corporations working with global populations in a technical environment: as a result, free online translation tools based on automatic translation (officially “machine translation" or MT), such as Google Translate and Microsoft Bing Translator, have rapidly gained popularity. These tools thus commonly meet the demand for translation coming from internet users who value speed, cost, and convenience over quality and who do not think professional translation services are necessary. Computer-aided translation (CAT) has, in turn, become prevalent in commercial translation production, and software solutions continue to dynamically change social communication in translation despite the translation industry remaining somewhat scattered and variable in its degree of sophistication regarding the use of such technologies [16].

Diaz [17] noted that an artificial intelligence robot, ChatGPT, created by OpenAI was released in November 2022. This was initially based on the GPT-3 series of big language models by OpenAI, though it has been improved using both supervised and reinforcement learning methods since its release. It was specifically created for use in conversation applications, including chatbots and messaging systems, being derived from a model from the GPT-3.5 series that completed training in early 2022.

Recent upgrades have led to the development of GPT-4, the most current step in OpenAI's deep learning scaling initiative. GPT-4 is a sizable multimodal model that accepts both picture and text inputs and emits text outputs. While less effective than humans in many real-world situations, GPT-4 performs at a human level on various tasks, based on the application of academic and professional benchmarks.

Artificial Intelligence Programs.

### 2.3 ChatSonic

ChatSonic is a creative AI writing assistant developed by Writesonic, which provides a range of AI-powered tools to help writers, marketers, and businesses create high-quality content. ChatSonic is designed to answer questions and write unique content such as blog posts, essays, and emails, and it uses natural language processing and machine learning algorithms to parse user requests and to use these to generate original, plagiarism-free content in real-time. ChatSonic was trained on a vast corpus of text data, allowing it to provide accurate answers to a wide range of questions across various fields of knowledge. This makes it a powerful tool for anyone looking to improve their writing skills, save time, or boost productivity. It also allows users to create text and images using voice commands, based on a powerful connection with Google search that helps it create hyper-relevant content and unique digital artwork and images [18].

#### 2.3.1 Bing Chat

Bing Chat is a new feature launched by Microsoft that uses AI technology to provide users with a “copilot for the web". It offers personalised recommendations, answers, and insights to users browsing the web, chatting with friends, or using the Edge browser or Bing, and Skype apps. Bing Chat is available on both Skype and Bing Mobile, and it is gaining popularity among users interested in AI-powered tools. However, available information on this AI is limited and a comprehensive overview of Bing Chat's features and capabilities has yet to be determined.

The new Bing chat assistant is also now available in a toolbar on the most recent stable version of Microsoft's Edge web browser. The function was previously only accessible as a developer beta, rather than a general release, having been first presented at Microsoft's AI press gathering in February. The “Edge Copilot" function was also available in Microsoft's beta release of the Microsoft Edge browser [19].

#### 2.3.2 ChatGPT

An artificial intelligence robot named ChatGPT, created by OpenAI, was released in November 2022, based on the GPT-3 series of big language models from OpenAI, for use in conversation apps such as chatbots or messaging systems. The application has been improved since then using both supervised and reinforcement learning methods, though a model from the GPT-3.5 series that completed training in early 2022 served as the basis for the system [17].

ChatGPT was recently upgraded to GPT-4, making this the most current step in OpenAI's deep learning scaling initiative. GPT-4 is a sizable multimodal model that accepts picture and text inputs and emits text outputs. While less effective than humans in many real-world situations, GPT-4 has been shown to perform at a human level according to various academic and professional benchmarks.

### 2.4 Machine language

Kenny [20] snooted that the emergence of statistical machine translation has been correlated with a new wave of technology-oriented studies in translation studies such as Jibreel (2023), Yang [11], Mahdy et al. [21], and Li et al. [12]. This makes it crucial to begin approaching the machine translation task using a formulation more in line with the task's actual complexity that thus reduces the distance to users' actual communication requirements. In the modern internet era, MT has become a lively field of practice and study, with such work having concrete repercussions for both individual members of the public and society as a whole, with benefits including emergency communication o achieve better rapid support during significant disasters [22]. According to Mahdy et al. [21], recent years have also seen rapid advancement in technology in the translation industry, commonly attributed to significant increases in task demand. Given the significant demand for rapid and precise translation, using machines in translation have become crucial. The quality of Neural Machine Translation (NMT) models has, however, recently increased, following developments in artificial intelligence, reducing the performance disparity between machine and human translation [13].

### 2.5 Technology and translation

Technology and translation are well paired, with translation working as a tool to allow worldwide contact in an increasingly globalised environment [15], facilitating user-centeredness. Translation practices have thus begun to integrate alternative situations based on the translation requirements of self-selected participants. Technological transformation has begun encroaching on various disciplines over time, particularly in the translation and cultural exchange fields, though the birth of machine translation depended on the massive development of artificial intelligence as created by the human mind. Constant growth in intelligent translation has allowed it to evolve significantly over recent decades to become more logical and closer to human translation,. According to Hartley [23], expectations for translation increased as the world moved into the digital age, however, and rather than relying exclusively on full, authoritative source texts, translation now often involves working with databases, glossaries, and electronic tools. Technology translation is thus a term that can be identified as referring to using any technology in translation, whether orally and verbally.

In the modern era, computerized formats have been applied to a range of creative text and other communication forms between customers and translators. These tools have had an essential impact on three areas: 1) communication (how translators interact with writers, customers, and other translators); 2) memory (how quickly and how much translators can recall); and 3) texts (as temporary arrangements of content). Odacıoglu and Kokturk [24] indicated that technological advancements assist the industry to flourish rapidly, and the development of computer-assisted translation tools such as translation memories, vocabulary databases, translation management applications, and electronic texts, particularly in the early 1980s, has changed how translators conduct the translation process. Wikipedia offers a good example examples of the effect of technology on translation. Wikipedia achieved success by engaging self-selected users to create an international encyclopaedia, supported by an army of volunteer interpreters. Wikipedia was originally only available in English when it launched in 2001, though the same year then saw the creation of the German, French, and Spanish sites in anticipation of wider international versions [25]. Arabic Wikipedia was then developed when translation teachers used it to educate students. Wikipedia material should commonly be translated into Arabic, according to Al Shehari Khaled [26], one of the foremost Arab language experts, yet while Wikipedia is helpful to translation projects, anyone can make and modify entries on Wikipedia, and only specific kinds of articles are subject to limitations.

### 2.6 Artificial intelligence applied to legal texts

Overall, the literature review underscores the pivotal role of AI in the realm of translation, particularly within the context of legal texts, suggesting that comparative analysis can illuminate the strengths and weaknesses inherent in both AI and human translation methodologies. However, it is evident that there are gaps in the current body of research. More specifically, there remains a need for more comprehensive studies that delve into the nuanced complexities of legal language and the capabilities of AI with respect to accurately capturing such subtleties. Furthermore, the potential for AI to learn and adapt to the intricacies of legal jargon and the implications of this for the field of translation remain largely unexplored avenues, suggesting that future research endeavours could focus on these areas in order to contribute to the evolving discourse on AI and human translation.

Machine translation, which uses artificial intelligence to speed up translation processes, has undoubtedly helped millions of people understand content in the form of texts in various languages. However, machine translation accuracy varies depending on the quality of the source text. Legal texts derived from such processes require accurate translation and understanding of nuances of meaning, as many legal terms have different uses in terms of form and content across types of text. To determine the degree to which the quality of a translation based on artificial intelligence matches that of a human translation in the legal context, this study applies technical analysis of the legal translation of various legal texts produced by artificial intelligence and compares these to human translations as performed by qualified translators.

## 3 Methodology

This study followed a qualitative method based on applying analysis and comparison strategies across nine steps, including the correcting, scoring, encoding, and decoding of data, and the use of analysis to answer the research questions.

### 3.1 Sample

Ten professional human translators took part, translating six legal texts. Three artificial intelligence programs were then used to translate the same legal texts.

#### 3.1.1 Sample selection

Ten professional human translators out of 30 possible options were selected from accredited translation agencies. All and had over five years of expertise in translating legal documents and good reputations in the translation market. Additionally, they were qualified based on their university degrees. Out of the 30 translators deemed suitable for this translation task, only ten were recruited, In terms of the selection of the intelligence programs, only three were chosen, which are intended to represent the pinnacle of intelligence programs as developed by the three globally recognised leading companies in this field.

#### 3.1.2 Selection of legal documents

Authentic legal documents were utilised on release by their business owners. These were redacted to hide the identities of the parties involved, at their request.

#### 3.1.3 Study validation

The validation process involved the selected certified legal documents being assessed by three expert translation professors to identify specific text segments to be used. The selection of human translators was based on their experience and reputation, and the evaluation and scoring process was then carried out by the three translation professors, including one who is a professional translator specialising in legal texts. Each assessor assessed each translation and assigned it a score ranging from one to four, based on specific criteria, as outlined in the separate file supplementary to this study.

Three well-known intelligence programs were used as mentioned in the literature review: GPT-4 is OpenAI's most advanced system, ChatSonic is the best ChatGPT alternative, and Microsoft Copilot has been recently developed by Microsoft, having been known as Bing Chat.

### 3.2 Limitations of the study

The sample size was relatively small because the legal document was long and took a long time to translate, limiting the number of translators. The choice of specific legal texts might also have introduced bias, as the selected texts may not represent the full range of complexity and diversity in legal language. The translation assessment process also risked various constraints, such as potential subjectivity in the evaluation of translation quality and difficulty in quantifying aspects such as style and tone.

### 3.3 Treatment

The process of treatment had two phases:

#### 3.3.1 Phase I

Several legal texts were selected from a variety of business contracts. After three translation professors validated the selected texts in terms of the approved translation, five professional legal translators were paid to ensure that the translation was both precise and expert. Three of the most well-known artificial intelligence systems were then given the same texts.

#### 3.3.2 Phase II

Three reputable assessors were passed the final translation production from both the artificial intelligence program and the human translators, along with the approved translation. They corrected and scored the translation performance of each and returned them to the researchers. The scores were then encoded and subjected to statistical analysis software to develop a quantitative comparison of the human group's translations and the translations produced by the artificial intelligences.

### 3.4 Procedures

Several procedures were used to conduct this study:
1. Writing the background of the study
2. Designing the study tools
3. Developing the methodology of the study
4. Selecting the legal texts
5. Validating the legal texts
6. Selecting artificial intelligence programs
7. Selecting professional legal translators
8. Correcting and scoring output translations
9. Analysing all data using a statistical program

## 4 Study hypotheses

There are no statistical differences between human and AI translations in Arabic.

There are no statistical differences between human and AI translations in English.

## 5 Data analysis

This section offers an analysis of the data collected from study participants in an attempt to answer the study questions. It thus comparatively evaluates the quality of translation between artificial intelligence translation and human translation.

Four approved criteria were used to evaluate the quality of the translation, based on approval by senior translation professors. Several criteria can be used to evaluate the quality of a translation: according to Ramos (2015), the model as used acts as a quality assurance model for legal translation, illustrating the potential benefits of enhancing predictability and reducing subjectivity for specific legal translation methodologies.

Table (1) shows the assessment quality translation criteria, adopted from Prieto Ramos [27] to evaluate the quality of legal translation. These five criteria were used to evaluate the legal translations by both human translators and AI.

1. Accuracy refers to the degree to which the translation effectively conveys the intended message from the original language to the desired language.
2. Translation competency pertains to the collection of skills that enable evaluation of the quality of a translation beyond mere success or failure.
3. Content should accurately convey the intended meaning of the source text.
4. Language, in the context of translation quality evaluation, pertains to the linguistic and non-linguistic elements that are part of the translation process.
5. Style in translation pertains to maintaining the stylistic elements of the source material in the translated content.

**Table 1:** Assessment quality translation criteria for legal translation.

Main Scales	Detailed Scales
**Accuracy**	Out of 20
**Competency**	Out of 20
**Content**	Out of 20
**Language**	Out of 20
**Style**	Out of 20
**Total**	**/100**

**Table 2:** Comparison of Translation Quality in Arabic Texts: Human vs. Artificial Intelligence Translations.

Scales	Human Translation			Mean	Artificial Intelligence			Mean
	Text 1	Text 2	Text 3		Text 1	Text 2	Text 3	
**Accuracy (20 Scores)**	16	19	19.3	**18.1**	17.3	17	17.3	**17.2**
**Competency (20 Scores)**	18.3	18.7	18.3	**18.4**	17.7	17	17	**17.2**
**Content (20 Scores)**	17.3	19.3	19.7	**18.8**	17.7	17.3	18	**17.7**
**Language (20 Scores)**	18	19.3	19	**18.8**	18.3	18	17.3	**17.9**
**Style (20 Scores)**	17.3	18.3	18.3	**18**	17.7	18.7	18.3	**18.2**
**Total (100)**	**87.3**	**94.7**	**94.7**	**92.2**	**88.7**	**88**	**88**	**88.2**

The professional translation text received was assigned a final rating of 100, indicating that the translation was perfect, with each criterion achieving 20 marks towards the final assessment. The scores for the five criteria for other versions were determined, based on the quality of translation, whether by a human or an artificial intelligence, using a statistical program. Only the outcomes are offered in this work, though comprehensive statistical procedure information and tables are provided in a separate attached file.

Table (2) shows a data analysis comparison between human translation and AI translation across three Arabic three texts, with five criteria used to evaluate these legal translations. The human translation mean score was 92.2, while the artificial intelligence translation scored 88.2.

Table (3) shows the data analysis comparison between human translation and AI translation in English for the three texts, based on the five criteria used to evaluate legal translations. The human translation mean score was 92.7, while the artificial intelligence translation scored 89.1.

Table 4 shows the overall comparison between human translation and AI translation in Arabic- English translations; the human translation mean score was 92.2, while the artificial intelligence translation scored 88.2.

**Table 3:** Comparison of data between human translation and AI translation in English translation texts.

**Scales**	Human Translation			Mean	Artificial Intelligence			Mean
	Text 1	Text 2	Text 3		Text 1	Text 2	Text 3	
**Accuracy (20 Scores)**	18.3	19.3	19	**18.9**	17.3	18	17.7	**17.7**
**Competency (20 Scores)**	18.7	18	18.7	**18.5**	17.3	17.3	17	**17.2**
**Content (20 Scores)**	17.7	19.3	18.3	**18.4**	18	17.3	17.7	**17.7**
**Language (20 Scores)**	19	18.7	19.7	**19.1**	18	17.7	18	**17.9**
**Style (20 Scores)**	17	18	18.3	**17.8**	19	18.7	18.3	**18.7**
**Total (100)**	**90.7**	**93.3**	**94**	**92.7**	**89.7**	**89**	**88.7**	**89.1**

**Table 4:** Overall comparison between English – Arabic human translation and artificial intelligence translation scores.

**Scales**	Human Translation	Artificial Intelligence	Comparison *P*-value
**Accuracy (20 Scores)**	18.1	17.2	**0.163**
**Competency (20 Scores)**	18.4	17.2	
**Content (20 Scores)**	18.8	17.7	
**Language (20 Scores)**	18.8	17.9	
**Style (20 Scores)**	18	18.2	
**Total (100)**	**92.2**	**88.2**	

Table 5 shows an overall data analysis comparison between human translation and AI translation for English-Arabic; the human translation mean score was 92.7, while the artificial intelligence translation scored 89.1.

**Table 5:** Overall comparison data between Arabic – English human translation and AI translation scores.

**Scales**	Human Translation	Artificial Intelligence	Comparison *P*-value
**Accuracy (20 Scores)**	18.9	17.7	**0.240**
**Competency (20 Scores)**	18.5	17.2	
**Content (20 Scores)**	18.4	17.7	
**Language (20 Scores)**	19.1	17.9	
**Style (20 Scores)**	17.8	18.7	
**Total (100)**	**92.7**	**89.1**	

Table 6 shows the overall comparison between the Arabic human translation mean (92.2) and the Arabic AI Translation mean (88.2) and the English human translation mean (92.7) and the English AI Translation mean (89.1).

**Table 6:** Overall mean comparison for Arabic and English between human translation and AI scores.

Human Translation (Arabic)	Artificial Intelligence (Arabic)	Comparison *P*-value
**92.2**	**88.2**	**0.163**
**Human Translation (English)**	**Artificial Intelligence (English)**	**0.240**
**92.7**	**89.1**	

## 6 Discussion

### 6.1 Assessment of human translation

**6.1.1. Arabic translation: The** human translators attempted to cope with the source using linguistic contradictions and discrepancies, as well as managing the usage of ordinary language, ; eading to the loss of some words' legal effect**.**

يتعهد الطرفان بالحفاظ على السرية التامة لكل المعلومات **التي يفصح كلٌ منهما للآخر** والتي **تتعلق بالعمل/التعامل التجاري** وفقاً لاتفاقية الوكالة **ولا يجوز لأي واحد** منهما الكشف عن هذه المعلومات فترة سريان العقد أو أثناء مدة ثمان سنوات **بعد انتهاء فترة العقد** إلا **إذا اقتضى القانون ذلك**.

**لا يتحمل أي طرف في هذا العقد المسؤولية الفشل في القيام بأحد بنود هذا العقد** بأي حال من الأحوال تجاه الطرف الآخر نتيجة لأي سبب طارئ عن الحرب، أو التمرد، أو الاضطراب، أو الإضراب، أو الإغلاق، أو النزاعات الصناعية، أو الحرائق، أو الانفجارات، أو الزلازل، **او ما قدر الله من أحداث خارج سيطرة** هذا طرف، كما يجب على هذا الطرف تقديم إشعاراً فورياً بذلك إلى الطرف الآخر. ويتحمل مسؤولية أي خسارة، أو ضرر، أو إصابة، أو أي نفقات يتكبدها الطرف الآخر لأسباب قاهرة كذه.

"المعرفة" هنا تعود إلى كل المعلومات التي يمتلكها (س) المتعلقة بالمنتجات واستخداماتها والتي **يتم إرسالها كتابياً إلى** (س**) خلال المدة المنصوص عليها في هذا العقد** والتي لا يعرفها (س) قبل وبعد مدة سريان والتي تعتبر ملكية عامة على سبيل المثال لا الحصر: الصيغة الطرق المستخدمة ومعلومات عن الصيغة والإجراءات ومراقبة الجودة والتقارير الفنية والخطط والمواصفات والملاحظات التي قد تكون مفيدة في استخدام وبيع المنتجات.

1. Assessment-based Accuracy: The translation was good, retaining the heart of the text and delivering meaning accurately generally. Some remarks were made regarding translating the terms the termination, Neither party hereto shall be under any liability, Act of God, and force majeure reason literally.
2. Assessment-based Competency: The Translation perfectly reflected the broad experience of the translator.
3. **Assessment-based Content:** the Content was clear and consistent.
4. ***Assessment-based* Language:***Correct legal terms were used in translation to reflect familiarity with legal effect.*
5. Assessment-based Style: A good style was used, based on the use of legal language and tone.

**6.1.2. English translation:** The human translators attempted to shade their translations to cope with the source. There were slight errors in the translation between the Arabic and the English text. The human translations offered good usage of legal terms, as well as correct use of legal phrases, sentence structure, and legal English language.

**Ex: Distribution agents must have prior written consent in order to represent**, manufacture, sell, or distribute products. **Directly or indirectly, distributors must not be involved in the manufacture**, sale, or even distribution of competing products.

If the distributor fails to meet the target at the end of any year **at least 90% of the minimum sales, the supplier may terminate this contract upon one month's advance notice.**

The owner grants and the distributer accepts by virtue of this agreement the exclusive right to **distribute the product in the region as long as it all conditions and rules stipulated in this agreement are met.** The distributor acknowledges and agrees that the rights awarded **by virtue of this agreement.** There is nothing in this agreement that prevents the owner to sell the products to any other person **outside of the region,** the distributor shall not either directly or indirectly distribute **or sell any product through any agents from outside of the region.**

1. Assessment-based Accuracy: The translation was good, preserving accuracy and consistency, though slight remarks were made regarding translating the terms: The owner grants and the distributer accepts by virtue of this agreement the exclusive right to, There is nothing in this agreement that prevents the owner, and distributors must not be involved in the manufacture literally.
2. Assessment-based Competency: The translation was generally good, despite missing some legal effects.
3. **Assessment-based Content:** the content was clear and close to the original
4. Assessment-based language: a lot of legal terms were missing in the translation, suggesting that the nature of legal language requires legal experience.
5. **Assessment based Style:** The style is good in terms consistency between texts and legal communication.

## 7 Comment

Human translation is often considered to be of higher quality due to the skill and expertise of the translator. A skilled human translator will deeply understand the languages and cultures they are translating between, making them able to accurately convey the intended meaning and tone of the original text. They can also pick up on subtleties and cultural nuances that may be lost in other forms of translation, using their judgment and knowledge to make informed decisions about translating idiomatic expressions, metaphors, and other language-specific features. This commonly results in a translation that accurately reflects the original text and is culturally appropriate for the target audience.

### 7.1 Part two: assessment of artificial intelligence

#### 7.1.1 Arabic translation

The AI translators attempted to cope with the source containing various linguistic contradictions and discrepancies, alongside handling the usage of ordinary language, leading to the loss of some words' legal effect**s**

Artificial Intelligence Translation:

تتعهد الأطراف أيضًا بالحفاظ على سرية جميع المعلومات المكشوفة من قبل بعضها البعض، فيما يتعلق بالأعمال التجارية بموجب اتفاقية الوكالة ولا يجوز لها في أي وقت خلال فترة الاتفاقية ولمدة إضافية تصل إلى ثماني سنوات بعد انتهائها كشف أي من هذه المعلومات لأي شخص، ما لم يكن ذلك مطلوبًا بموجب القانون.

لا يتحمل أي من الطرفين المتعاقدين أية مسؤولية تجاه الطرف الآخر بأي شكل من الأشكال في حالة عدم القدرة على تنفيذ أي شرط من هذا الاتفاق بسبب أي سبب ينشأ عن حالة الحرب، التمرد، الاضطراب، الإضراب، إغلاق المصانع أو نزاع صناعي، حرائق، انفجارات، زلازل، وأسباب طبيعية خارجة عن سيطرة هذا الطرف. وسوف يقوم بإخطار الطرف الآخر بشكل فوري بهذه المشكلة ولا يتحمل أية مسؤولية تجاه أية خسائر، أو أضرار، أو إصابات أو نفقات يتعرض لها الطرف الآخر بسبب هذه الظروف التي لا يمكن التحكم فيها.

"المعرفة الفنية" تعني المعلومات التي تمتلكها XXXX المتعلقة بالمنتجات واستخدامها، والتي يتم نقلها بالكتابة إلى XXXXXX خلال الفترة المحددة هنا، ولا يعرفها XXXXXX قبل أو بعد ذلك وتعد ملكًا عامًا، ولكن ليس محصورًا على ذلك، بما في ذلك الصيغة، وطرق الاستخدام، والمعلومات حول الصيغة، والإجراءات وضبط الجودة، والتقارير التقنية، والخطط، والمواصفات، والملاحظات التي قد تكون مفيدة أثناء استخدام المنتجات وبيعها."

1. Assessment-based Accuracy: The three AI translations failed to interpolate the accurate meaning of missing words, and lacked legal terms, using alternative words such as بعضها البعض, الاعمال التجارية, لايتحمل أي من الطرفين المتعاقدين أي مسؤولية,مالم يكن ذلك مطلوباً بموجب القانون
2. Assessment based Competency: The translation lacked competency, translating “business” as الاعمال التجارية، سرية المعلومات المكشوفة،, لا يتحمل أي من الطرفين المتعاقدين أية مسؤولية تجاه الطرف الآخر
3. Assessment-based Content: The were some missing words and mismatches with words such as the use of “secret” for “confidential”
4. Assessment-based Language: The language used was relatively clear, with minor inconsistencies regarding the rendering of meaning in the target language
5. Assessment based Style: The style missed out on offering a legal effect based on the use of simple language instead of legal language: بعد انتهائها, ما لم يكن ذلك مطلوبًا بموجب القانون., يتم نقلها بالكتابة إلى, هذه الظروف التي لا يمكن التحكم فيها

**6.2.2. English Translation:** The AI translators tried to cope with variations in the source in their translation. However, there was a severe mismatch between the Arabic text and the English translation. The three AI translations had various similarities in terms of the usage of exact words and the sentences, as well as the use of legal phrases, sentence structurse, and plain English.

The distributor is not allowed to represent, manufacture, sell, or distribute in the region or anywhere else unless he obtains written approval from the source. (Or directly or indirectly participate in the manufacture, sale, or distribution of competing products) during the entire term of this contract. He must also not engage in work as an agent for the distributor,

If a distributor fails at the end of any year to achieve at least 90% of the minimum sales target, the supplier has the right to terminate this contract upon prior notice of one month. However, he has the right to submit a written letter within two months after the end of the year in which the above-mentioned goal was not achieved, indicating the reasons.

This agreement grants the owner and distributor the exclusive right to distribute the product in the region, subject to all terms and conditions set forth in this agreement. The distributor agrees to purchase the product exclusively from the owner for their own account and solely market, distribute, and sell this product in the region. The distributor acknowledges that the rights granted under this agreement are limited to this region,

1. Assessment based Accuracy: The three AI translations failed to determine the accurate meaning of missing words and lacked legal terms, using alternative simple words such as لا يجوز للموزع تمثيل, كما يجب عليه ألا ينخرط, إذا فشل موزع في نهاية أي سنة في تحقيق ما لا يقل عن 90%, ويقر ويوافق الموزع على أن الحقوق الممنوحة بموجب هذا الاتفاق محصورة في هذا الإقليم
2. Assessment based Competency: The translation lacks competency in translating phrases such as يمنح المالك ويقبل الموزع بموجب هذه الاتفاقية الحق الحصري لتوزيع المنتج في الإقليم على أن تخضع ،, يحق للمورد إنهاء هذا العقد بناء على أشعار مسبق مدته شهر
3. Assessment-based Content: Several crucial legal phrases were lost, while others were substituted ineffectively. Based on this, certain legal implications have not been fully understood.
4. Assessment-based Language: The language is relatively clear, despite mismatches in some legal phrases
5. Assessment-based Style: The English style does not have the required legal tone to set it apart as legal English used in contracts. Legal language was missing in several phrases. The model verbs such as shall and may, which are essential in legal language, are not used in the artificial intelligence translations, which are written in a professional writing-style using simple English.

#### 7.1.2 Comment

AI translation quality positively influences legal content, and proper knowledge beyond the content of texts is required to achieve successful translation. Even minor translation mistakes can have significant consequences, as legal terminology is frequently intricate and precise; thus, while AI translation has advanced significantly in recent years, producing translations quickly and affordably, it might not be best suited to capture the nuances and complexities of legal language faithfully.

The human translation was found to be more accurate and to come nearer to the intended meaning, although the AI translations were found to be generally correct and sound, despite slight variations in word choice. In terms of contrasting the accuracy of translations produced by humans and AI, very little significant difference emerges:

### 7.2 Similarities and differences

6.3.1. Human Translations:

#### 7.2.1 Strengths

1. Understanding of Context: Human translators are able to understand and interpret the context, cultural nuances, and idiomatic expressions contained in the source language, which can be challenging for AI.
2. Handling Ambiguity: Humans are better at handling ambiguous phrases and sentences, using their knowledge and understanding of the world to infer the correct meanings.

Example: Where the English phrase “**Act of God**" appears, a human translator would know that this is a legal term used to refer to a natural disaster, especially prior to contract performance, and would translate this accordingly in the target language.

##### 7.2.1.1 Weaknesses

1. Speed and Scalability: Human translations are time-consuming and may not be feasible for large volumes of text.
2. Consistency: There may be inconsistencies in translations done by different human translators or even by the same translator at different times.

Example: The English term “Act of God" can have multiple meanings; hus, depending on the context, a human translator might translate it differently at different times, leading to inconsistency.

#### 7.2.2 AI translations

##### 7.2.2.1 Strengths

**Speed and Scalability**: AI can translate large volumes of text quickly, making it suitable for onerous tasks such as translating web pages or books.

**Consistency**: AI translations are consistent, being based on fixed algorithms and databases.

Example: An AI might be trained to translate the English phrase “Act of God” into the Arabic term for “natural disaster" in a legal context.

##### 7.2.2.2 Weaknesses

Understanding of Context: AI struggles with understanding and interpreting context, cultural nuances, and idiomatic expressions in the source language.

Handling Ambiguity: AI may not handle ambiguous phrases and sentences well, as it cannot infer meaning based on its own knowledge.

Example: If an AI encounters the English phrase “Act of God" without prior training, it might translate this literally, missing the idiomatic meaning.

AI and human translations both have strengths and weaknesses with respet to translating text from one language to another. AI-based translation systems can translate text quickly and accurately, leveraging advanced machine learning algorithms and natural language processing techniques. However, these systems may struggle with complex linguistic structures, idioms, and cultural references. On the other hand, while human translators have a deep understanding of the languages they translate and can accurately convey the original text's meaning and intent, as well as easily handling complex linguistic structures and cultural references, human translation can be time-consuming, as well as being less efficient than AI-based translation systems. In recent years, there has thus been a growing interest in combining the strengths of AI and human translation to create hybrid systems that can more accurately and efficiently translate text. These systems leverage the speed and accuracy of AI-based translation while incorporating the expertise and understanding of human translators.

In terms of the quality of translation, there are some critical differences between human and AI translation. Human translators deeply understand the languages and cultures they are translating between and can accurately convey the intended meaning and tone of the original text: they can also pick up on subtleties and cultural nuances that may be lost in AI translation.

Overall, both AI and human translation offer advantages and disadvantages, and the best approach in each case will depend on the specific needs and requirements of the translation task at hand.

According to Murphy [28], formal assessment of translation quality is essential to recognise the difference between mistakes and preferential choices, however, as translators will make informed decisions based on the target audience and any contextual material they have been given to help them understand a company's tone and voice. Based on the current work, the results of data analysis for the translation texts for both human and AI translators showed that the human translation scored more highly for both the Arabic translation (92.2) and the English translation (92.7) in comparison with the AI translation, which scored only 88.2 for the Arabic translation and 89.1 for the English.

This indicates a slight superiority in human translation over AI translation in terms of legal work; the human translation is superior because it can be based on knowing the legal background and be distinguished by maintaining legal effect. The translation by AI may improve in the future, based on the advancement of technology and the increase in data entered into the program; however,to date, the translations by AI are all similar and lack legal effect, instead utilising simple English.

The mean scores in the tables offer a quantitative measure of the quality of both types of translations. Higher mean scores suggest better translation quality in a given area: for instance, a higher mean score in accuracy indicates that the translation is more faithful to the source text. Similarly, a higher mean score in fluency suggests that the translation reads more naturally in the target language.

The significance of the differences between the mean scores of human and AI translations was determined using appropriate statistical tests. Where a p-value is less than the chosen significance level (0.05), the difference is considered statistically significant. In this case, this implies that there is a significant difference in the quality of the translations produced by humans and AI.

Regarding the hypotheses proposed, that there is no statistically significant difference between the human translation and the AI translation in both Arabic and English, the results of the study demand that these be rejected. The results of the study do not support the initial hypothesis, with the evidence gathered during the study indicating a difference in outcomes on analysis.

While the hypothesis suggested no statistically significant differences in accuracy between human and AI translations in Arabic would exist, where any difference does exist, it might be expected that human translations would be more accurate due to the complex nature of the Arabic language, which might be challenging for AI to fully grasp. Similarly, if a difference exists between translation types into English, it might be expected for the AI translations to be less fluent, as AI might not fully capture the nuances and idiomatic expressions common in the English language.

Based on the discussion above, the study's most significant findings are as follows:

• Due to the specialist translators' broad practical experience and theoretical and legal backgrounds, human translation can be distinguihed from AI translation.
• Notwithstanding the similarities between all three translations produced by the three artificial intelligence-derived algorithms chosen for this study, artificial intelligence translation has undergone remarkable improvement in terms of understanding legal documents. Nonetheless, these systems could not comprehend the text's full meaning and the intricate legal background thereby represented.
• With the advancement of data and logarithmic rules for artificial intelligence in the future, it may be possible to achieve 100% scores in specialised legal translation. This might require ab artificial intelligence translation program to be trained on all existing agreements and legal texts used, which might allow it to eventually replace human translation in terms of efficiency, language proficiency, and text understanding, using appropriate synonyms and translations in each case.

These findings provide valuable insights into the comparative performance of human and AI translators, particularly in terms of accuracy and fluency. Further research could thus delve deeper into these aspects in the attempt to optimise the translation process and improve overall translation quality.

## 8 Conclusions

Human translation and AI translation (also known as machine translation) are two different approaches to translating text from one language to another. Human translation should be performed by a person fluent in both the source and target languages who profoundly understands the cultural context and nuances of the text. AI translation, however, is performed by a computer program using algorithms and large amounts of data to translate the text. While AI translation can be faster and more cost-effective than human translation, owever, it may not always capture the subtleties and cultural nuances of the text as accurately as a human translator can.

While artificial intelligence translation has made great strides in recent years and can provide fast and cost-effective options, it still has many limitations. Human translation, on the other hand, offers a deeper understanding of the cultural context and nuances of the translated text: a skilled human translator can accurately convey the intended meaning and tone of the original text, making this a superior choice for translations that require a high level of accuracy and cultural sensitivity. As legal terminology and concepts can vary between different jurisdictions, AI translation tools may not always be able to account for these differences. Currently, it is thus recommended that a skilled human translator with expertise in legal translation is used to ensure the highest levels of accuracy and quality when translating legal texts.

As AI Translation relies on algorithms and large amounts of data to translate text, while it can provide fast and cost-effective translations, it may not always capture the subtleties and cultural nuances of the text as accurately as a human translator may. Additionally, AI Translation may struggle with idiomatic expressions, metaphors, and other language-specific features that a human translator is likely to understand and translate accurately.

While AI translation has some advantages, human translation may thus be considered to provide higher quality translation due to its increased ability to accurately convey the original text's intended meaning and cultural context. As this study focused on the legal field, however, further extensive research in artificial intelligence is needed to address this issue in the fields of medicine, politics, religion, and journalism.

## Ethical approval

Review and/or approval by an ethics committee was not needed for this study as it did not involve collecting data from human participants. All translation texts were pre-existing, having been created by paid human translators or by legal use of AI translators. This does not require ethical approval from an institutional review board (IRB), as all use of pre-existing translation texts complies with copyright laws and proper credit was given to the human translators.

## Funding

After publishing a research paper about Artificial Intelligence and human translation, the authors may seek potential funding from the University of Bisha for grants for further research. Having a published paper can help demonstrate the authors’ expertise and the value of their work, making it more likely that they will be successful in securing funding.

## Availability of data and materials

The authors affirm that the data used in the study is available in the form of an appendix file. Any other researchers are thus allowed to access the data and verify the results of the study. There are no conditions or restrictions on the use of the data, and there is no need to obtain permission from the authors.

## CRediT authorship contribution statement

**Ahmed Mohammed Moneus:** Writing – original draft. **Yousef Sahari:** Writing – review & editing, Validation, Project administration, Formal analysis.

## Declaration of competing interest

The authors of this paper declare that they have no conflicts of interest in relation to the research presented in this paper. The research was conducted independently and was not funded by any organisation with a vested interest in the results. The authors have no financial or personal relationships with any individuals or organisations that could potentially influence the research.

## Figures and Tables

**Table:** The Analysis Criteria

Main Scales	Detailed Scales
**Accuracy**	20 Scores
**Competency**	20 Scores
**Content**	20 Scores
**Language**	20 Scores
**Style**	20 Scores
**Total**	**100 Scores**

Analysis Of Arabic Translation.

**Table:** Human Translation

Scales	HT1				HT2				HT3			
	Text 1	Text 2	Text 3	Mean	Text 1	Text2	Text 3	Mean	Text1	Text 2	Text3	Mean
**Accuracy (20 Scores)**	16	15	17		19	20	18		19	19	20	
**Competency (20 Scores)**	***18***	***19***	***18***		***19***	***19***	***18***		***18***	***18***	***19***	
**Content (20 Scores)**	17	18	18		19	20	19		19	20	20	
**Language (20 Scores)**	18	17	19		19	19	20		20	18	19	
**Style (20 Scores)**	16	18	18		18	18	19		19	18	18	
**Total (100)**	85	87	90		94	96	94		95	93	96	

Analysis Of English Translation.

**Table:** Human Translation

Scales	HT1				HT2				HT3			
	Text 1	Text 2	Text 3	Mean	Text 1	Text2	Text 3	Mean	Text1	Text 2	Text3	Mean
**Accuracy (20 Scores)**	18	18	19		19	19	20		18	19	20	
**Competency (20 Scores)**	***19***	***19***	***18***		***18***	***18***	***18***		***19***	***18***	***19***	
**Content (20 Scores)**	16	18	19		19	20	19		16	19	20	
**Language (20 Scores)**	19	19	19		18	19	19		19	20	20	
**Style (20 Scores)**	17	17	17		18	18	18		17	20	18	
**Total (100)**	89	91	92		92	94	94		89	96	97	

Analysis of Arabic Translation.

**Table:** Artificial Intelligence Translation

Scales	AI1				**AI 2**				AI 3			
	**Text 1**	**Text 2**	Text 3	Mean	Text 1	Text 2	Text 3	Mean	Text 1	Text 2	Text 3	Mean
**Accuracy (20 Scores)**	17	16	19		17	16	18		17	17	18	
**Competency (20 Scores)**	18	17	18		17	17	17		17	17	17	
**Content (20 Scores)**	17	18	18		18	17	17		18	18	18	
**Language (20 Scores)**	18	18	19		18	18	18		17	17	18	
**Style (20 Scores)**	17	17	19		19	18	19		18	18	19	
**Precentage (100)**	**87**	**86**	**93**		**89**	**86**	**89**		**87**	**87**	**90**	
**Mean**												

Analysis of English Translation.

**Table:** Artificial Intelligence Translation

Scales	AI1				**AI 2**				AI 3			
	**Text 1**	**Text 2**	Text 3	Mean	Text 1	Text 2	Text 3	Mean	Text 1	Text 2	Text 3	Mean
**Accuracy (20 Scores)**	18	17	17		17	19	18		18	18	17	
**Competency (20 Scores)**	17	17	18		17	18	17		17	17	17	
**Content (20 Scores)**	18	18	18		18	17	17		18	17	18	
**Language (20 Scores)**	18	18	18		18	18	17		18	18	18	
**Style (20 Scores)**	19	19	19		19	19	18		19	19	17	
**Precentage (100)**	**90**	**89**	**90**		**89**	**89**	**87**		**90**	**89**	**87**	
**Mean**												

**Table:** Comparison Between Human Translation And Artificial Intellgience In Arabic Translation Texts

Scales	Human Translation			Mean	Artificial Intelligence			Mean
	Text 1	Text 2	Text 3		Text 1	Text 2	Text 3	
**Accuracy (20 Scores)**								
**Competency (20 Scores)**								
**Content (20 Scores)**								
**Language (20 Scores)**								
**Style (20 Scores)**								
**Total (100)**								

**Table:** Comparison Between Human Translation And Artificial Intelligence In English Translation Texts

Scales	Human Translation			Mean	Artificial Intelligence			Mean
	Text 1	Text 2	Text 3		Text 1	Text 2	Text 3	
**Accuracy (20 Scores)**								
**Competency (20 Scores)**								
**Content (20 Scores)**								

**Table:** Overall Comparison Between (English – Arabic) Human Translation And Artificial Intelligence Translation Scores

Scales	Human Translation	Artificial Intelligence	Comparison
**Accuracy (20 Scores)**			
**Competency (20 Scores)**			
**Content (20 Scores)**			
**Language (20 Scores)**			
**Style (20 Scores)**			
**Total (100)**			

**Table:** Comparison between (Arabic – English) Human Translation and Artificial Intelligence Translation Scores

Scales	Human Translation	Artificial Intelligence	Comparison
**Accuracy (20 Scores)**			
**Competency (20 Scores)**			
**Content (20 Scores)**			
**Language (20 Scores)**			
**Style (20 Scores)**			
**Total (100)**			
